# The Synthesis of Copper Nanoparticles for Printed Electronic Materials Using Liquid Phase Reduction Method

**DOI:** 10.3390/ma17133069

**Published:** 2024-06-21

**Authors:** Kai Li, Xue Jiang

**Affiliations:** College of Bioresources Chemical and Materials Engineering, Shaanxi University of Science & Technology, Xi’an 710021, China; likai824123591@163.com

**Keywords:** printed electronics, copper nanoparticles, sintering, conductive ink, ascorbic acid reduction method

## Abstract

This text discusses the synthesis of copper nanoparticles via a liquid phase reduction method, using ascorbic acid as a reducing agent and CuSO_4_·5H_2_O as the copper source. The synthesized copper nanoparticles are small in size, uniformly distributed, are mostly between 100–200 nm with clear boundaries between particles, and exhibit excellent dispersibility, making them suitable for metal conductive inks. 1. The copper nanoparticles are analyzed for good antioxidation properties, because their surface is coated with PVP and ascorbic acid. This organic layer somewhat isolates the particle surface from contact with air, preventing oxidation, and accounts for about 9% of the total weight. 2. When the prepared copper nanoparticles are spread on a polyimide substrate and sintered at 250 °C for 120 min, the resistivity can be as low as 23.5 μΩ·cm, and at 350 °C for 30 min, the resistivity is only three times that of bulk copper. 3. The prepared conductive ink, printed on a polyimide substrate using a direct writing tool, shows good flexibility before and after sintering. After sintering at 300 °C for 30 min and connecting the pattern to a circuit with a diode lamp, the diode lamp is successfully lit. 4. This method produces copper nanoparticles with small size, good dispersion, and antioxidation capabilities, and the conductive ink prepared from them demonstrates good conductivity after sintering.

## 1. Introduction

The rapid development of internet and information technology has indeed provided fertile ground for the emergence of various new concepts, among which the Internet of Things (IoT) is a notable example [1]. The IoT enables objects to collect and exchange data by installing sensors and other intelligent devices on them, facilitating intelligent control and management. This process heavily relies on the development of electronic components [2,3]. Printed electronic technology, as a new type of electronic component manufacturing technology, offers a low-cost, efficient manufacturing solution by directly printing circuits and devices on flexible materials or paper using conductive inks [4,5]. This technology is particularly suitable for the production of large-area, flexible electronic products. It plays a significant role in the development of IoT because it can significantly reduce the cost of intelligent devices, making more objects capable of being smart [6,7].

In the field of conductive inks, nano-metallic conductive inks have attracted widespread attention due to their excellent electrical conductivity, low sintering temperatures, and good flexibility [8,9,10]. Gold and silver nanoparticles were the focus of early research, but their high cost limits large-scale application [11,12,13]. Bok Yeop Ahn and colleagues used silver nitrate as a precursor, polyacrylic acid as a capping agent, and diethanolamine as a reducing agent to obtain silver nanoparticles with an average size of 20 ± 5 nm. These silver nanoparticles are highly dispersible in water and organic solvents, forming conductive ink, which is then used to form conductive grids through a direct writing method [14]. Huang and colleagues synthesized silver nanoparticles with an average diameter of 30–50 nm, using monoethanolamine as a reducing agent and PAA (polyacrylic acid) as a capping agent for inkjet printing. After printing the ink on a photo paper substrate and sintering, the resistivity of the printed silver film could reach 8.0 × 10^−6^ Ω·cm after drying at room temperature. By increasing the sintering temperature to 180 °C, the resistivity of the printed silver patterns could be reduced to approximately 3.7 × 10^−6^ Ω·cm [15]. Moscick and colleagues prepared silver nanoparticles with a size distribution of 50–70 nm using PVP (polyvinylpyrrolidone) as a surface modifier for the silver particles, with sodium citrate acting as the reducing agent. The prepared silver particles were used as conductive fillers, and a mixed solution of ethanol and glycol was used as the solvent to formulate the conductive ink. After printing into circuits and curing at 150 °C for 60 min, the resulting printed silver circuit had a resistivity of approximately 5 × 10^−5^ Ω·cm [16]. In contrast, copper nanoparticles have become a research hotspot, due to their low cost and good electrical conductivity [17,18,19,20]. Bong Kyun Park and colleagues used copper sulfate as a precursor, polyvinylpyrrolidone (PVP) as a capping agent, sodium hypophosphite as a reducing agent, and diethylene glycol (DEG) as a solvent to prepare copper particles with an average size of 45 ± 8 nm at 140 °C. They made copper conductive ink using a mixed solution of glycol and methoxyethanol as the solvent [21]. Zhang Yu and colleagues from the Shenzhen Institutes of Advanced Technology, Chinese Academy of Sciences, used copper hydroxide as a precursor, polyethylene glycol as a capping agent, ascorbic acid as a reducing agent, and ethylene glycol as a solvent to prepare antioxidant, monodisperse copper nanoparticles, with a size of 135 ± 30 nm. They applied a conductive paste containing methyl cellulose, butyl glycol ether, and copper nanoparticles onto a polyimide substrate via screen printing. After thermal treatment at 250 °C for 30 min, they obtained a sintered pattern with a resistivity of 15.8 μΩ·cm [22]. However, the biggest challenge with copper nanoparticles is their propensity to oxidize, which can affect electrical conductivity and long-term stability. Therefore, developing copper nanoparticles with strong antioxidation capabilities has become a key scientific task [23]. Addressing the oxidation issue of copper nanoparticles often involves the development of surface modification and protective coatings, such as using organic, inorganic, or polymeric materials as surface protectants to prevent the copper particles from coming into contact with oxygen and moisture in the air [24,25,26]. These methods aim to not only enhance the antioxidation properties of copper nanoparticles but also maintain their good electrical conductivity and processability for better application in printed electronics [27,28]. Therefore, researching and developing copper nanoparticles with high antioxidation capabilities is a crucial driving force for the advancement of printed electronic technology and IoT. As the related technologies continue to progress and mature, the future application scenarios of IoT will become more extensive, device costs will decrease, and the level of smartness will be enhanced.

This study aims to explore how to enhance the antioxidative properties of copper nanoparticles while ensuring they possess excellent electrical conductivity and flexibility. Our specific method involves using ascorbic acid for reduction and coating the surface of the copper nanoparticles with polyvinylpyrrolidone. This technique is intended to improve the overall performance and stability of the copper nanoparticles through surface modification.

## 2. Materials and Methods

### 2.1. Chemicals

In this study, the materials used, including copper sulfate pentahydrate (CuSO_4_·5H_2_O), polyvinylpyrrolidone (PVP K30), ascorbic acid, anhydrous ethanol, ethylene glycol, and ethyl cellulose, were all purchased from Aladdin Reagent Co., Ltd., Shanghai, China. They were used as received without the need for further purification.

### 2.2. Preparation of Copper Nanoparticles

1. CuSO_4_·5H_2_O is weighed and dissolved in ethylene glycol at room temperature, with magnetic stirring, to prepare a copper source precursor solution with a concentration of 0.1 M;
2. Ascorbic acid is weighed and dissolved in ethylene glycol at room temperature, with magnetic stirring, to prepare a reducing agent precursor solution with a concentration of 0.2 M;
3. PVP is weighed and dissolved in ethylene glycol at 150 °C, with vigorous magnetic stirring, to prepare a coating agent precursor solution with a concentration of 0.1 M;
4. The copper source precursor solution and the reducing agent precursor solution obtained in steps 1 and 2 are co-added to the PVP precursor solution from step 3 at a rate of 1 mL/min, and the mixture is stirred in an oil bath at 150 °C for 120 min;
5. After the reaction is completed, the reaction system is allowed to cool down to room temperature naturally, and the resulting product is washed three times with ethanol and deionized water at a speed of 9000 rpm, then dried under vacuum at 40 °C.

### 2.3. Preparation of Conductive Ink

In the first set of experiments, 1.8 g of ethanol, 0.2 g of copper nanoparticles, and varying amounts of PVP (0.03 g, 0.06 g, 0.09 g), along with 0.1 g of ethyl cellulose, were used. The mixture containing copper particles was stirred for 10 min and ultrasonically dispersed for 30 min to observe the precipitation situation.

In the second set of experiments, ethanol was replaced with ethylene glycol, while other conditions remained unchanged.

The third set of experiments varied the solvent composition across six groups: 1.6 g ethanol/0.2 g ethylene glycol, 1.3 g ethanol/0.5 g ethylene glycol, 1.0 g ethanol/0.8 g ethylene glycol, 0.8 g ethanol/1.0 g ethylene glycol, 0.5 g ethanol/1.3 g ethylene glycol, and 0.2 g ethanol/1.6 g ethylene glycol, with 0.06 g of PVP used in each group, and other conditions kept constant.

By comparing the three groups of experiments, the group with the best dispersion was selected as the optimal formula for the ink.

### 2.4. Homemade Conductive Ink Pen

**Figure 1:**
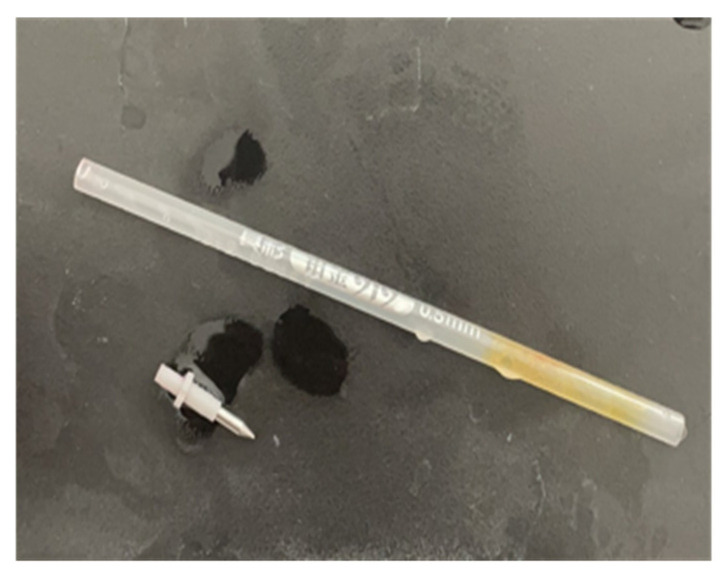
Homemade conductive ink pen. (ZHENCAI, A brand of ink pens made in China).

An experiment was conducted to create a homemade conductive ink pen. The ink cartridge of a commonly used sign pen was taken and rinsed with distilled water to remove the ink inside. After the ink was thoroughly washed out, anhydrous ethanol was used to rinse again to remove any residual ink. Then, the ink cartridge and pen tip were soaked in anhydrous ethanol and ultrasonically cleaned. After cleaning for 20 min, the ink cartridge and pen tip were taken out, dried with air, and reassembled (Figure 1).

### 2.5. Electrical Properties of Copper Nanoparticles after Sintering

Copper nanoparticles prepared by the liquid phase reduction method need to exhibit good electrical properties, especially conductivity, when used for printing electronic components. An appropriate sintering temperature can promote the formation of sintering necks among copper nanoparticles, establishing conductive pathways and reducing resistivity, thus enabling the functionality of electronic components. We use a homemade conductive pen to apply copper ink onto polyimide films, followed by sintering. The electrical performance of the sintered copper nanoparticles is primarily assessed by their resistivity; the lower the resistivity, the better the conductivity and the better the performance of the electronic components. Since the resistivity of copper nanoparticles is related to the sintering temperature and time, we will adjust these two parameters in our experiments to measure the resistivity under different conditions.

### 2.6. Analysis and Characterization

The microstructure and morphology of the samples were observed using a Scanning Electron Microscope (SEM; VEGA 3 SBH, Tescan, Brno, Czech Republic); the chemical structure of the samples was determined by X-ray Photoelectron Spectroscopy (XPS; AXIS SUPRA, Manchester, UK) and X-ray Diffraction (XRD; D8 Advance, BRUKER, Karlsruhe, Germany). The elemental composition of the samples was analyzed with an X-ray Energy Dispersive Spectrometer (EDS; VEGA 3 SBH, Tescan, Brno, Czech Republic); and the thermal stability of the samples was analyzed using a Thermogravimetric Analyzer (TGA; STA449F3, Netzsch, Selb, Germany).

## 3. Results

### 3.1. Characterization of Copper Nanoparticles

**Figure 2:**
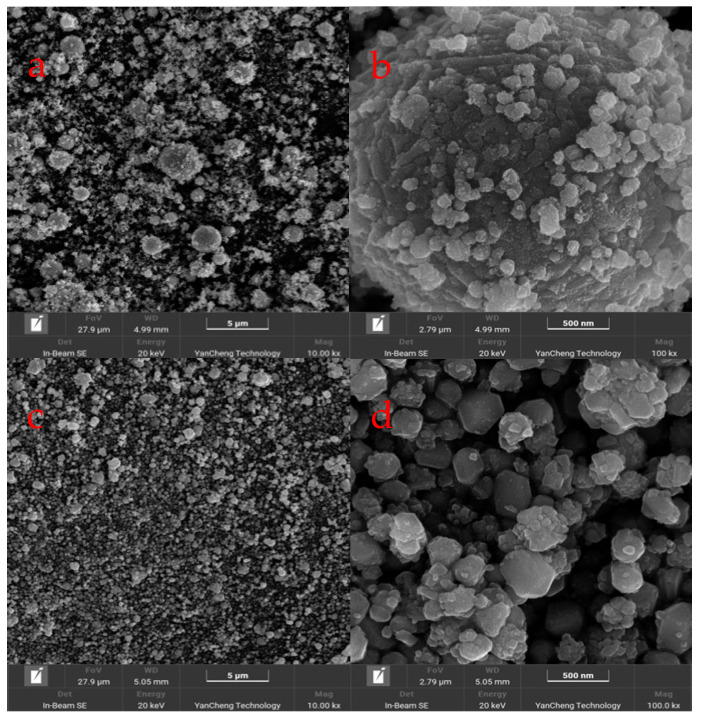
SEM Images of copper nanoparticles: (**a**) Low magnification SEM image of the purchased copper nanoparticles (**b**) High magnification SEM image of the purchased copper nanoparticles (**c**) Low magnification SEM image of the synthesized copper nanoparticles (**d**) High magnification SEM image of the synthesized copper nanoparticles.

We used SEM (Scanning Electron Microscopy) to observe the detailed surface morphology of the samples (Figure 2).

**Figure 3:**
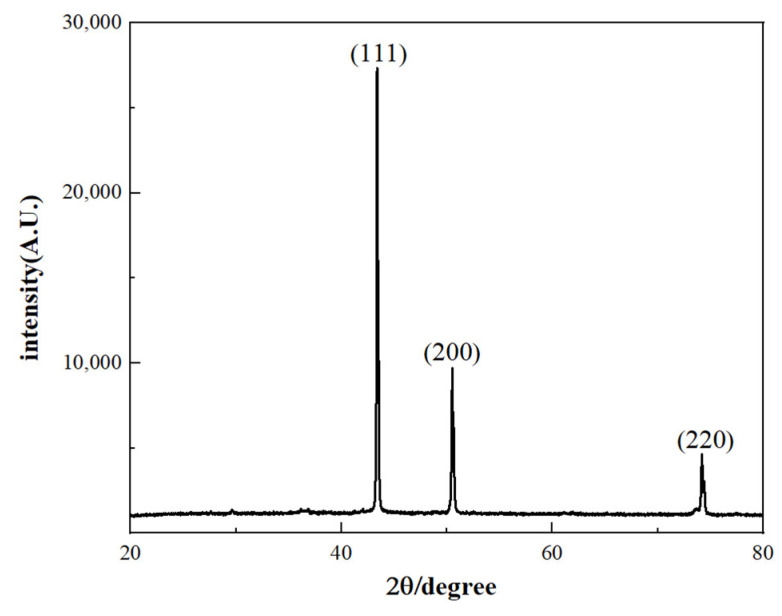
XRD Spectrum of copper nanoparticles.

The X-ray diffractometer is an analytical instrument that irradiates a sample with X-rays and then detects the diffraction phenomenon produced by the scattering of atoms within the sample. By analyzing the diffraction results, information about the sample’s crystal structure can be obtained (Figure 3). Observing the XRD result chart allows us to understand the crystal plane structure of copper nanoparticles and determine the presence of copper oxide.

**Figure 4:**
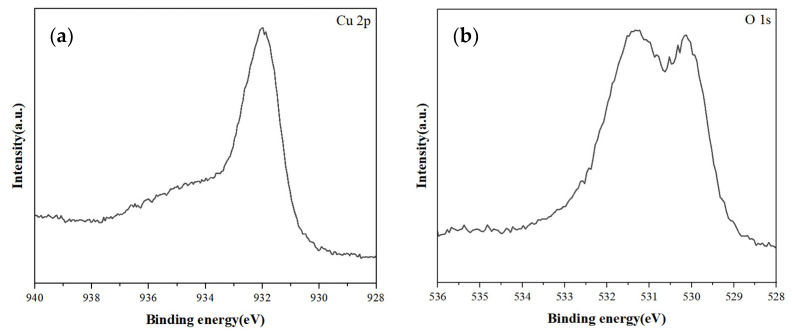
XPS Spectrum of copper nanoparticles: (**a**) Cu 2p, (**b**) O 1s.

Figure 4 displays the XPS spectra of copper and oxygen in the sample. From Figure 4a, it can be observed that the Cu 2p peak of the prepared copper particles is located at a binding energy of 932.3 eV, corresponding to the peak of pure copper, while the Cu 2p peak for copper oxide is at approximately 934.3 eV binding energy.

**Figure 5:**
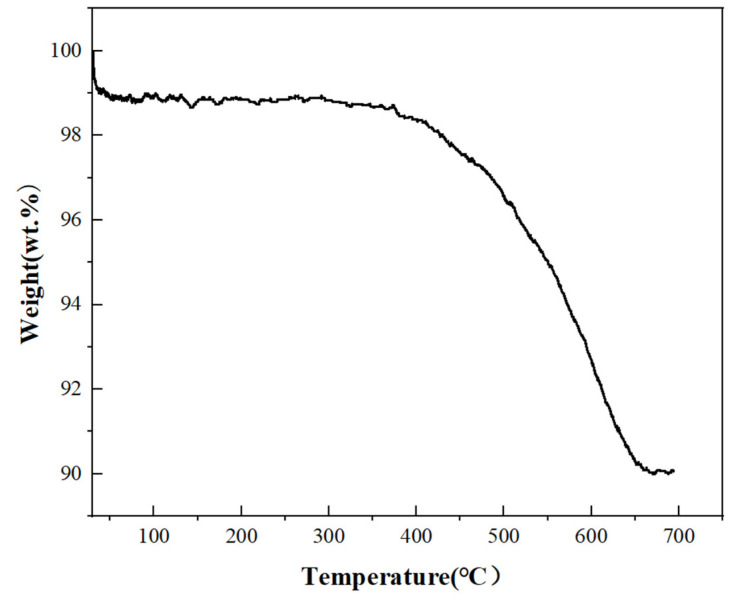
The thermogravimetric analysis spectrum of copper nanoparticles.

Thermogravimetric analysis is a technique used to measure the relationship between the mass of the sample and temperature changes. In the experiment, 3 mg of the sample was placed into a small crucible, and heated from room temperature to 700 °C at a rate of 10 °C/min under nitrogen protection (Figure 5). From the graph, it is evident that before the temperature reached 100 °C, the sample’s mass decreased to approximately 98.8%, which includes the residual small amounts of water, ethanol, and ethylene glycol, accounting for about 1.2% of the total. Upon further heating to 400 °C, the sample’s mass decreased slowly, and upon heating to 650 °C, the sample’s mass sharply decreased to at least 90%.

**Figure 6:**
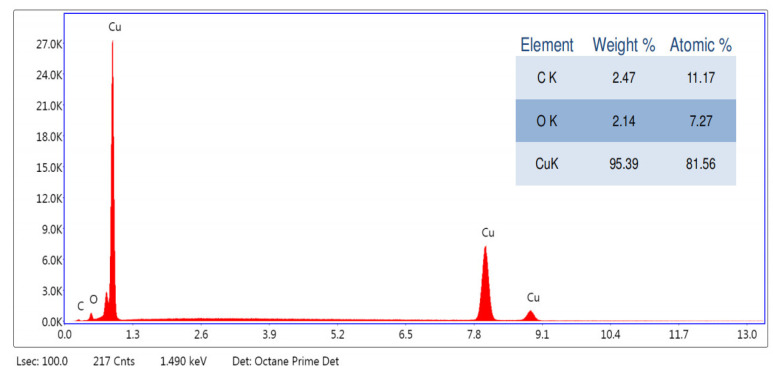
The energy spectrum analysis spectrum of copper nanoparticles.

An energy spectrometer uses X-rays to irradiate a sample, where each element within the sample has its characteristic wavelength. Based on the different characteristic energies, the type and content of elements can be obtained (Figure 6).

### 3.2. Preparation of Conductive Ink

**Figure 7:**
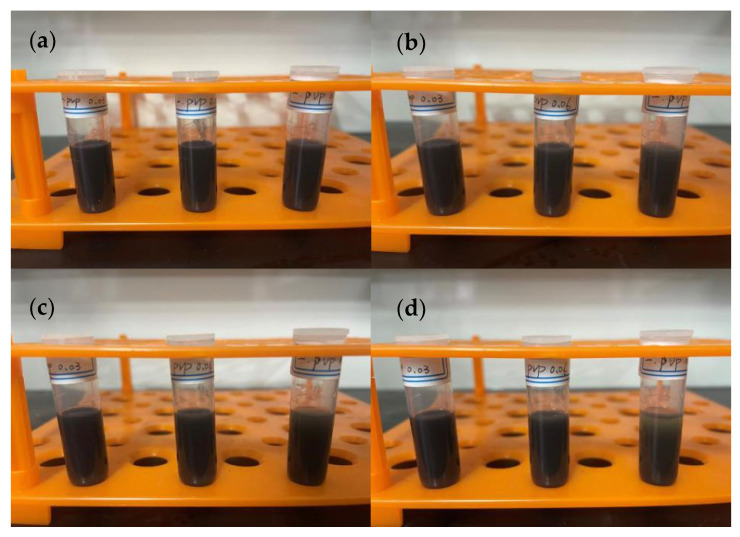
Using ethanol as the solvent, with PVP amounts from left to right being 0.03 g, 0.06 g, and 0.09 g, respectively: (**a**) after standing for 0 h, (**b**) after standing for 8 h, (**c**) after standing for 16 h, (**d**) after standing for 24 h.

**Figure 8:**
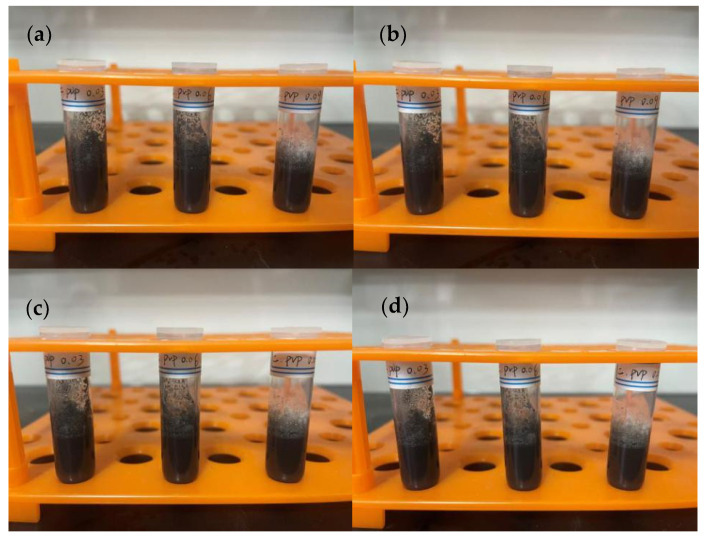
Using ethylene glycol as the solvent, with PVP amounts from left to right being 0.03 g, 0.06 g, and 0.09 g, respectively: (**a**) after standing for 0 h, (**b**) after standing for 8 h, (**c**) after standing for 16 h, (**d**) after standing for 24 h.

**Figure 9:**
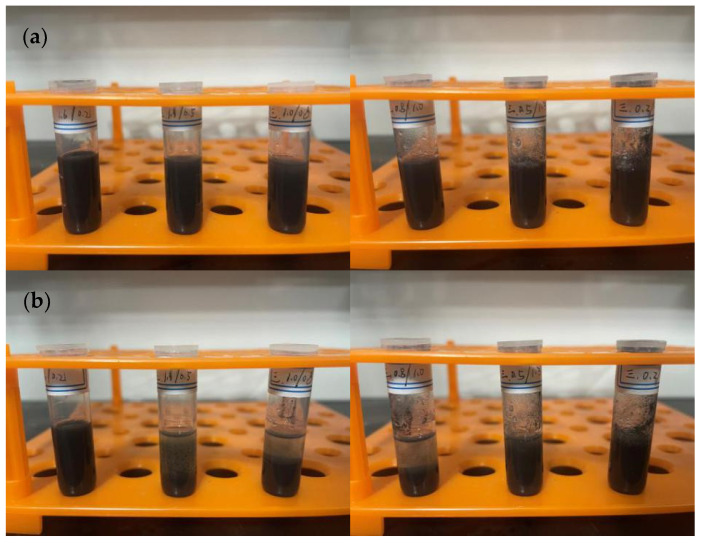
Using 0.06 g of PVP, the solvent composition ratios from left to right are as follows: ethanol 1.6 g/ethylene glycol 0.2 g; ethanol 1.3 g/ethylene glycol 0.5 g; ethanol 1.0 g/ethylene glycol 0.8 g; ethanol 0.8 g/ethylene glycol 1.0 g; ethanol 0.5 g/ethylene glycol 1.3 g; and ethanol 0.2 g/ethylene glycol 1.6 g. (**a**) Static for 0 h, (**b**) Static for 8 h.

**Figure 10:**
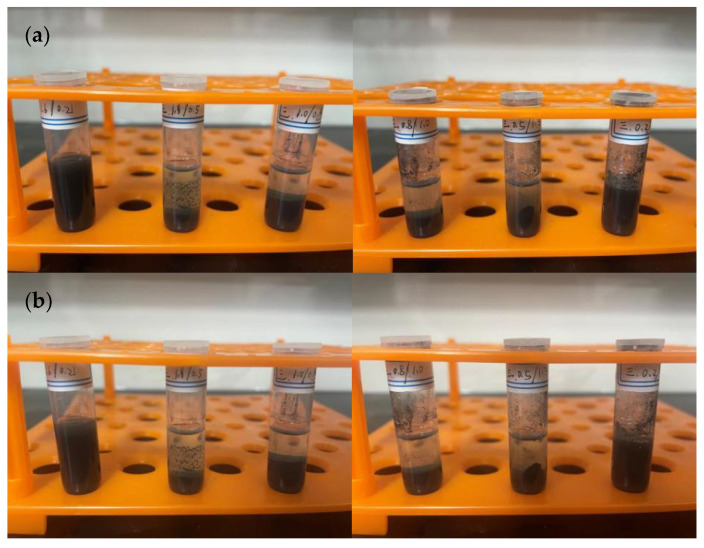
Using 0.06 g of PVP, the solvent composition ratios from left to right are as follows: ethanol 1.6 g/ethylene glycol 0.2 g; ethanol 1.3 g/ethylene glycol 0.5 g; ethanol 1.0 g/ethylene glycol 0.8 g; ethanol 0.8 g/ethylene glycol 1.0 g; ethanol 0.5 g/ethylene glycol 1.3 g; and ethanol 0.2 g/ethylene glycol 1.6 g. (**a**) Static for 16 h, (**b**) Static for 24 h.

Most of the inks on the market use volatile organic solvents with good dispersion as carriers. In this setup, ethanol, ethylene glycol, and their mixture were chosen as solvents, PVP as the dispersant, and ethyl cellulose as the binder. In the first set of experiments, 1.8 g of ethanol, 0.2 g of copper nanoparticles, and varying amounts of PVP, along with 0.1 g of ethyl cellulose, were used. The solution containing copper particles was stirred for 10 min and ultrasonically dispersed for 30 min to observe the precipitation (Figure 7). In the second set of experiments, ethanol was replaced with ethylene glycol, keeping the other conditions unchanged (Figure 8). In the third set of experiments, just the quantities of ethanol and ethylene glycol were changed, with 0.06 g of PVP used in each group, keeping the other conditions constant (Figure 9 and Figure 10).

### 3.3. Electrical Properties of Copper Nanoparticles after Sintering

The resistivity of copper nanoparticles is influenced by the sintering temperature and sintering time. Therefore, in this experiment, we primarily adjusted these two parameters to measure resistivity. The selected sintering temperatures were 200 °C, 250 °C, 300 °C, and 350 °C, while the sintering time was determined by observing when the sheet resistance no longer showed significant changes. The measurement of sheet resistance was conducted using an RTS-8 type four-point probe tester, and the relationship between the measured results and the sintering temperature and time is presented in Table 1.

The resistivity of the film is calculated based on the product of the sheet resistance and the film thickness. Due to the thickness variability of the films applied by doctor blading, it is necessary to measure the thickness of each sample. The presence of raised edges in the doctor-bladed samples results in a higher surface roughness compared to the film thickness; therefore, the resistivity of the copper films is calculated based on the estimated thickness of the flat part of the film. The calculation of resistivity is derived from the average of multiple samples, and the specific results are presented in Table 2.

**Figure 11:**
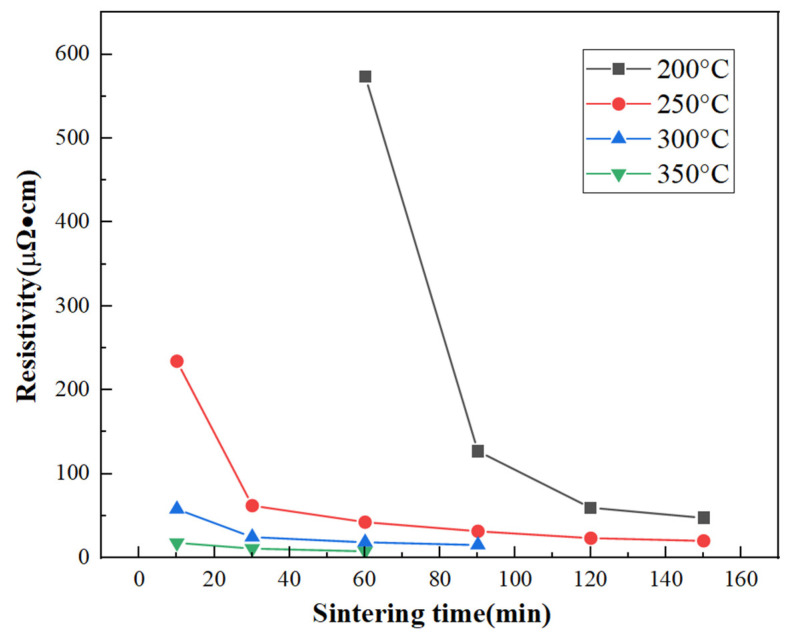
The trend chart of copper film resistivity under different sintering temperatures and sintering times.

**Figure 12:**
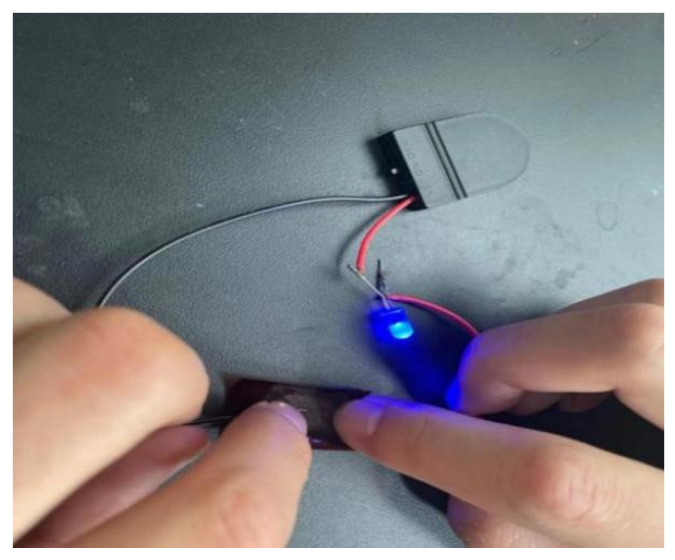
Illustration of polyimide film coated with conductive ink sintered at 300 °C.

To more clearly explore the trend of resistivity changes with sintering temperature and time, we plotted Figure 11. The figure shows that at lower sintering temperatures, the resistivity sharply decreases with time until it stabilizes. In contrast, at higher temperatures, the change in resistivity is relatively slow. At the same sintering temperature, the resistivity gradually decreases with the extension of sintering time until it stabilizes. After sintering for 30 min at 300 °C, the polyimide film coated with the ink with optimal dispersibility successfully lights up a diode lamp connected in the circuit (Figure 12). We purchased commonly available wires, batteries, and light-emitting diodes on the market, connected them, and connected the ends of the wires to a polyimide film to test whether a circuit could be formed. After sintering at 300 °C for 30 min, we connected the polyimide film coated with the ink with optimal dispersibility to the circuit, turned on the switch, and found that the diode lit up. The battery used here is a 3 V button battery, and combined with the previously tested resistance, we determined that our experiment was successful. The light-emitting diode could be lit up using a small voltage power source, indicating that the copper nanoparticles formed a good sintered neck between them after appropriate temperature sintering, connecting the particles together to form a complete circuit. This will be very helpful for the future of large-scale flexible electronic printing.

## 4. Discussion

### 4.1. Characterization of Copper Nanoparticles

In Figure 2a, the purchased particles are magnified 10,000 times, revealing their uneven size distribution. At 80,000 times magnification, the particles appear as irregular spheres (Figure 2b). In contrast, Figure 2c shows the homemade copper nanoparticles magnified 10,000 times, where their size distribution is more uniform, and they exhibit better dispersibility. At 80,000 times magnification, the boundaries of the homemade particles are clearly observable, with shapes being irregular, some appearing as thin flakes or rods, and most sizes ranging between 100–200 nm (Figure 2d).

The X-ray diffractometer is an analytical instrument that irradiates a sample with X-rays and then detects the diffraction phenomenon produced by the scattering of atoms within the sample (Figure 3). In this spectrum, we can observe three distinct and sharp peaks at angles 2θ = 43.3°, 50.6°, and 74.7° [29]. These angles correspond to the crystal planes (111), (200), and (220), respectively, which match the standard spectrum of pure copper (Figure 3). Thus, it can be seen that the copper nanoparticles obtained by reducing copper sulfate pentahydrate with ascorbic acid do not contain copper oxide or cuprous oxide. This indicates that the influence of polyols and organic coating agents during the preparation process endows the copper nanoparticles with antioxidative properties, thereby producing pure copper without other phases.

Figure 4 displays the XPS spectra of copper and oxygen in the sample. From Figure 4a, it can be observed that the Cu 2p peak of the prepared copper particles is located at a binding energy of 932.3 eV, corresponding to the peak of pure copper, while the Cu 2p peak for copper oxide is at approximately 934.3 eV binding energy. Additionally, an XPS analysis was conducted on oxygen. After the deconvolution of the original spectrum, three peaks were obtained, corresponding to the binding energy positions of 530.3 eV, 531.9 eV, and 533.4 eV. These correspond to the binding energy of oxygen in the carbonyl group without bonded copper atoms, the binding energy of oxygen in the carbonyl group bonded with copper atoms, and the binding energy of oxygen in the hydroxyl group, respectively. The XPS spectra can be quantitatively analyzed to a certain extent. From the areas of the three peaks in the figure, it can be seen that the area of oxygen in the hydroxyl group accounts for about 20% of the total area. The solvent DEG, containing hydroxyl groups, was almost completely washed away, so the hydroxyl groups come from ascorbic acid. Therefore, it can be inferred that the surface of the synthesized copper nanoparticles contains ascorbic acid. Since it only accounts for 20%, most of it is still due to the bonding of oxygen atoms in PVP and copper atoms, so most of the surface of the copper particles is still PVP. Due to the combined effect of PVP and excess ascorbic acid, it inhibits the growth of copper nanoparticles and to a certain extent isolates the contact between the oxygen in the air and the surface of copper nanoparticles, making them exhibit certain antioxidant properties.

Thermogravimetric analysis is a technique used to measure the relationship between the mass of the sample and temperature changes. As shown in Figure 5, before the temperature rises to 100 °C, the mass of the sample slowly decreases to 98.8%, which includes a very small amount of residual water, ethanol, and ethylene glycol, accounting for about 1.2% of the total. Continuing to heat up to 400 °C, the mass of the sample slowly decreases, and when the temperature reaches 650 °C, the mass is reduced to a minimum of 90%. During this process, the most significant is the decomposition and evaporation of ascorbic acid and PVP, which account for about 8% of the weight. After that, when the temperature rises to 700 °C, the total mass very slowly increases. This is because the organic coating on the surface of the copper nanoparticles has evaporated. Although there is nitrogen protection, the sealing of the instrument itself and the purity of the nitrogen cause the copper nanoparticles to undergo slight oxidation.

An energy spectrometer uses X-rays to irradiate a sample, where each element within the sample has its characteristic wavelength. The energy spectrum and table in Figure 6 display the results for copper nanoparticles in the area irradiated by X-rays. The energy spectrum reveals that the sample contains three elements: Cu, C, and O, with Cu being the most abundant, and distributed in three positions. The energy spectrometer can also perform a quantitative analysis to a certain extent. According to the table, Cu accounts for 95.39% of the total weight, which, considering the influence of cleaning fluids and solvents, is essentially consistent with the thermogravimetric analysis results. The table shows an atomic ratio of C to O as 11.17:7.27. Given that the atomic ratio of C to O in PVP is 6:1 and in ascorbic acid is 1:1, it can be inferred that the sample contains both PVP and ascorbic acid. Therefore, copper nanoparticles, prepared using an excess of ascorbic acid to reduce copper sulfate pentahydrate and coated with both PVP and ascorbic acid, exhibit good antioxidative properties during both the reaction and storage processes, providing a solid foundation for their application in various fields.

### 4.2. Preparation of Conductive Ink

According to Figure 7, it can be observed that changing the amount of PVP in the ethanol solvent does not significantly affect the dispersion of particles. Even after 24 h, no significant precipitation or clearing of the solution was observed, allowing for flexibility in the choice of PVP amount. According to Figure 8, when ethylene glycol is used as the solvent, significant precipitation occurs after 8 h, and with an increase in dispersant, the degree of precipitation slightly decreases. After 24 h, solutions with a small amount of PVP nearly completely precipitate, while those with more PVP still have minimal precipitation, indicating that ethylene glycol alone is not suitable as a solvent for conductive ink. According to Figure 9 and Figure 10, using a mixture of ethanol and ethylene glycol results in poor dispersion. Among all mixtures, the best effect shown in Figure 10b is for ethanol 1.6 g/ethylene glycol 0.2 g, and the worst is for ethanol 0.8 g/ethylene glycol 1.0 g. It appears that a small amount of ethanol can improve the dispersion of copper particles in ethylene glycol, but a slight excess leads to poor results. Through comparative experiments, the ink composition was finalized, including ethanol 1.8 g, copper nanoparticles 0.2 g, PVP 0.06 g, and ethyl cellulose 0.1 g. Once prepared, the ink exhibited good dispersibility and viscosity, maintaining excellent dispersion even after being left undisturbed for 24 h.

### 4.3. Electrical Properties of Copper Nanoparticles after Sintering

The performance of electronic components largely depends on the electrical properties of copper nanoparticles after sintering, where resistivity is an important metric. A lower resistivity value indicates better conductivity, and thus, better performance in electronic components. The resistivity of copper nanoparticles is influenced by the sintering temperature and sintering time. Therefore, in this experiment, we primarily adjusted these two parameters to measure resistivity. The selected sintering temperatures were 150 °C, 200 °C, 250 °C, 300 °C, and 350 °C, while the sintering time was determined by observing when the sheet resistance no longer showed significant changes. The measurement of sheet resistance was conducted using an RTS-8 type four-point probe tester, and the relationship between the measured results and the sintering temperature and time is presented in Table 1. According to the data in Table 2, it can be observed that even at a temperature of 150 °C and a sintering time of up to 150 min, the resistivity of the copper film could not be measured. This suggests that at 150 °C or even lower temperatures, regardless of the sintering duration, copper nanoparticles are still unable to form sintering necks, and thus, no electronic channels exist, preventing the material from exhibiting conductivity. On the other hand, based on the thermogravimetric analysis results, the decomposition and evaporation of organic matter begin to be observed at 180 °C. Therefore, at 150 °C, the surface of the copper nanoparticles is still covered by PVP and ascorbic acid, making it impossible to exhibit conductivity. This finding is consistent with the conclusions from the thermogravimetric analysis.

When the temperature is raised to 200 °C, the resistivity of the copper films begins to stabilize after sintering for 120 min, at around 59.8 μΩ·cm. At a temperature of 350 °C, it only takes 10 min of sintering for the resistivity to drop to 18.2 μΩ·cm, reaching a stable value of 8.1 μΩ·cm after 60 min. This result is more favorable compared to previous reports in the literature.

To more clearly explore the trend of resistivity changes with sintering temperature and time, we plotted Figure 11. The main reasons for this phenomenon include: Increased Conductivity [30]: during the sintering process, sintering necks form between copper nanoparticles, leading to decreased resistivity, thereby improving conductivity, which allows the diode lamp in the circuit to be lit. Stable Conductive Pathways [31]: during the sintering process, the organic coatings gradually decompose and evaporate, exposing the copper particle surface and forming stable conductive paths, which aids in the flow of current through the circuit to light the diode lamp.

Appropriate Temperature and Time [32]: after sintering for 30 min, the polyimide film at 300 °C may have reached an optimal state, making the connections between copper nanoparticles sufficiently stable while avoiding excessive charring or damage to the film, thereby achieving a good conductive performance.

In summary, after sintering for 30 min at 300 °C, the polyimide film coated with ink of optimal dispersibility successfully lights up a diode lamp connected in the circuit (Figure 12). This is mainly because the sintering process improved the material’s conductivity and established stable conductive pathways, while the choice of temperature and time brought the film’s performance to a good balance point (Figure 12).

## 5. Conclusions

We proposed a simple yet effective method for synthesizing copper nanoparticles from copper sulfate pentahydrate via liquid phase reduction and further applying them in the preparation of conductive ink for the fabrication of flexible electronic devices. In this study, we arrived at several key conclusions:

Firstly, using ascorbic acid as a reducing agent, we successfully synthesized copper nanoparticles that are small in size and exhibit good dispersibility, with most particles ranging in size from 120–160 nm. These particles have clear boundaries and are suitable for the preparation of metal conductive ink. Through various characterization methods, we discovered that the surface of the copper nanoparticles is coated with PVP and ascorbic acid. This organic coating layer somewhat prevents contact between the particles and the air, thereby endowing the particles with good antioxidation properties. The weight of this organic coating layer accounts for about 9% of the total weight.

Secondly, through comparative experiments, we determined the formula for conductive ink with the best dispersibility and applied it to a polyimide substrate. The coated patterns showed good flexibility before and after sintering, maintaining excellent performance even after the sintering process. Notably, after sintering at 300 °C for 30 min, the pattern coated with conductive ink was connected to a circuit with a diode lamp, successfully lighting the diode lamp. This demonstrates the practical application potential of conductive ink in electronic components.

Finally, we tested the resistivity of samples sintered at different temperatures. The results showed that the resistivity could be reduced to 23.5 μΩ·cm after sintering at 250 °C for 120 min, and to only three times that of bulk copper after sintering at 350 °C for 30 min. These findings indicate that the conductive ink we prepared has good conductive performance, and by adjusting the sintering temperature and time, different conductive properties can be achieved, providing important references for the fabrication of flexible electronic devices.

## Figures and Tables

**Table 1:** The sheet resistance (unit: mΩ/sq) of copper thin films at different sintering temperatures and sintering times.

	10 min	30 min	60 min	90 min	120 min	150 min
200 °C	-	-	956.9	208.2	100.7	80.9
250 °C	398.3	105.9	72.4	53.6	40.8	35.4
300 °C	99.7	42.8	31.3	26.9	-	-
350 °C	32.4	20.1	15.5	-	-	-

**Table 2:** The resistivity (unit: μΩ·cm) of copper thin films at different sintering temperatures and sintering times.

	10 min	30 min	60 min	90 min	120 min	150 min
200 °C	-	-	574.1	127.1	59.8	47.8
250 °C	234.5	62.3	42.6	31.9	23.5	20.5
300 °C	58.2	24.9	18.5	15.5	-	-
350 °C	18.2	11.2	8.1	-	-	-

## Data Availability

The data supporting the article’s findings are available from the corresponding author upon reasonable request.
